# Comparison of conventional and modern methods in determining ischemic stroke etiology by general and stroke neurologists

**DOI:** 10.3906/sag-1806-29

**Published:** 2019-02-11

**Authors:** Refik KUNT, Mustafa Kürşad KUTLUK, Bedile İrem TİFTİKÇİOĞLU, Nazire AFŞAR, Ali Kemal ERDEMOĞLU, Muhteşem GEDİZLİOĞLU, Vesile ÖZTÜRK

**Affiliations:** 1 Clinic of Neurology, Aydın State Hospital, Aydın Turkey; 2 Department of Neurology, Faculty of Medicine, Dokuz Eylül University, İzmir Turkey; 3 Department of Neurology, Faculty of Medicine, Başkent University, İzmir Zübeyde Hanım Medical and Research Center, İzmir Turkey; 4 Department of Neurology, Faculty of Medicine, Acıbadem University, İstanbul Turkey; 5 Clinic of Neurology, Ankara Koru Hospital, Ankara Turkey; 6 Department of Neurology, Bozyaka Education and Research Hospital, İzmir Turkey

**Keywords:** TOAST, CCS, ischemic stroke, etiology

## Abstract

**Background/aim:**

This study aimed to investigate the consistency between stroke and general neurologists in subtype assignment using the Trial of ORG-10172 in Acute Stroke Treatment (TOAST) and Causative Classification of Stroke (CCS) systems.

**Materials and methods:**

Fifty consecutive acute ischemic stroke patients admitted to the stroke unit were recruited. Patients were classified by two stroke and two general neurologists, each from different medical centers, according to TOAST followed by the CCS. Each neurologist was assessed for consistency and compliance in pairs. Concordance among all four neurologists was investigated and evaluated using the kappa (ĸ) value.

**Results:**

The kappa (ĸ) value of diagnostic compliance between stroke neurologists was 0.61 (95% CI: 0.45–0.77) for TOAST and 0.78 (95% CI: 0.62–0.94) for CSS-5. The kappa (ĸ) value was 0.64 (95% CI: 0.48–0.80) for TOAST and 0.75 (95% CI: 0.60–0.91) for CCS-5 for general neurologists. Compliance was moderate [ĸ: 0.59 (95% CI: 0.52–0.65)] for TOAST and was strong [ĸ: 0.75 (95% CI: 0.68–0.81)] for CCS-5 for all 4 neurologists. ‘Cardioembolism’ (91.04%) had the highest compliance in both systems. The frequency of the group with ‘undetermined etiologies’ was less in the CCS (26%) compared to TOAST.

**Conclusion:**

The CCS system improved compliance in both stroke and general neurologists compared with TOAST. This suggests that the automatic, evidence-based, easily reproducible CCS system was superior to the TOAST system.

## 1. Introduction

Ischemic stroke (IS) is one of the most common heterogeneous diseases known to be caused by multiple potential etiologies and can occur in many different etiological combinations in parallel with advances in diagnostic technologies (1,2). It is not possible to recognize and treat such a complicated disease without using a functional classification system. In addition, a functional classification system is indispensable for patient selection for clinical trials, phenotyping, and evaluation of prognosis for genetic and epidemiological studies (2).

TOAST (Trial of ORG-10172 in Acute Stroke Treatment) and the CCS (Causative Classification of Stroke) are two well-known systems for classifying IS (Table 1) (3–7). TOAST, which is one of the traditional classification systems and has been used for over 20 years without losing its importance, gives no idea about what plays the predominant role when there is more than one etiological cause. Almost half of the stroke patients are assigned to the ‘undetermined etiologies’ group by the TOAST system when more than one possible etiology is defined as the stroke mechanism (4–6). Indeed, IS can often be the final result of multiple abnormalities, and treatment decisions require a more comprehensive assessment, such as that provided by the CCS (8). The CCS, one of the modern classification systems, is a semiautomatic classification system that is freely available to anyone with an Internet connection (6). The main objective of the CCS is to reduce the limitations of the TOAST system (i.e. to reduce the rate of the unclassified group). It was also developed to improve interclass reliability in IS classification by providing interclinician language cohesion in interpreting stroke-related features (2,4–6).

**Table 1:** TOAST and CCS classification systems and subgroups.

TOAST	CCS		
	5 main subtypes	8 main subtypes	16 subtypes
Large artery atherosclerosis	Supraaortic Large artery atherosclerosis	Supraaortic Large artery atherosclerosis	Supraaortic Large artery atherosclerosis
Probable - Possible			Evident - Probable - Possible
Cardiac embolism	Cardioaortic embolism	Cardioaortic embolism	Cardioaortic embolism
Probable - Possible			Evident - Probable - Possible
Small vessel occlusion	Small arterial occlusion	Small arterial occlusion	Small arterial occlusion
Probable - Possible			Evident - Probable - Possible
Other reasons detected	Other reasons detected	Other reasons detected	Other reasons detected
Probable - Possible			Evident - Probable - Possible
Undetermined	Undetermined	Undetermined	Undetermined
	Cryptogenic embolism	Cryptogenic embolism	Cryptogenic embolism
Idiopathic	Other cryptogenic	Other cryptogenic	Other cryptogenic
Incomplete evaluation	Incomplete evaluation	Incomplete evaluation	Incomplete evaluation
Unclassified(multiple etiologies)	Unclassified (multiple etiologies)	Unclassified (multiple etiologies)	Unclassified (multiple etiologies)

The CCS distributes ischemic stroke into 5 major etiologic groups such as the TOAST system (CCS-5). If the ‘Undetermined’ group is evaluated by distribution into three subgroups in the CCS, there will be 8 subgroups for the CCS (CCS-8). If the other four major subgroups are defined at three points, which are obvious, probable, and possible, according to the weight of causal evidence (the risk of primary stroke associated with each cause), the CCS-16 system may be mentioned. Thus, stroke mechanisms are arranged in order of greatest to smallest and the most possible cause of stroke can be found (4–6).

In studies involving multiple international centers, it was stated that the CCS indicated a high level of harmonization among evaluators (1,2,4–7,9–11). We aimed to investigate the concordance between evaluators who classify IS using either TOAST or the modern CCS system. Since, in general, highly experienced evaluators are investigated in IS studies, we aimed to create heterogeneity among the evaluators by including both stroke specialists (stroke neurologists) and general neurology specialists (general neurologists) who have less experience but still manage stroke patients in their general neurology practice.

## 2. Materials and methods 

### 2.1. Study population

A total of 50 consecutive acute IS patients admitted to and registered in the Dokuz Eylül University Hospital Stroke Unit were recruited prospectively into the study, following the approval of the local ethics committee. 

### 2.2. Data collection

During the hospitalization of the patients, medical records were registered as data files generated by two local staff in the stroke unit and a registrar. These files included demographic data (age, sex, etc.) and medical history, as well as the neurological examination and results of radiological, cardiological, and serum biochemical tests of the patients.

The medical history included queries about hypertension (a history of hypertension or an observed arterial blood pressure of >140/90 mmHg), diabetes mellitus (presence of a history of diabetes mellitus or a fasting glucose exceeding 126 mg/dL other than that measured during the acute phase), hyperlipidemia (positive history of hyperlipidemia or a fasting total cholesterol >200 mg/dL, LDL >130 mg/dL, and/or triglycerides >180 mg/dL), smoking habits, alcohol consumption, previous transient ischemic attack or stroke, myocardial infarction or coronary artery disease, cardiac valvular disease, cardiomyopathy or cardiac rhythm disorders, peripheral vascular diseases, oral contraceptives, or hormone replacement therapy. Detailed neurological examination and NIHSS (National Health Institute Stroke Scale) (12) and modified Rankin Scale (mRS) (13) scores were also recorded. The results of complete blood count, fasting serum blood glucose, liver and renal function tests, serum electrolytes, lipid profile, and levels of vitamin B12 and folic acid were noted. Analysis of coagulation factors (protein C, protein S, antithrombin III, prothrombin II, and factor V Leiden), and advanced vasculitis examinations (lupus anticoagulants, anticardiolipin antibody, antinuclear antibodies, anti-DNA, antineutrophil cytoplasm antibodies) were performed in selected cases. Brain parenchyma (computerized tomography, CT; magnetic resonance imaging, MRI) and vascular imaging (CT angiography, CTA; magnetic resonance angiography, MRA; Doppler ultrasonography, Doppler US; digital subtraction angiography, DSA) were performed according to the clinical status of the patient. The results of neuroimaging were registered as reported by the radiodiagnostic department. All radiological images were stored digitally. Electrocardiography (ECG) was performed in all patients, but Holter ECG and echocardiography (transthoracic, TTE and transesophageal, TEE) were performed only if indicated to investigate the cardiac risk factors. 

### 2.3. Procedure

A detailed patient data file was created for each case. This file included digital neuroimages stored in separate folders on a USB flash memory stick, as well as neurological examination with NIHS score and results of cardiac and biochemical tests. These were delivered to 2 stroke neurologists highly experienced in cerebrovascular neurology and 2 general neurologists who manage stroke patients within their routine general neurology practice. All raters were from different neurology clinics, unaware of the other evaluators and the reference opinion. The reference opinion, from someone who did not participate in patient care during this period, was the final decision made by the highly experienced head of the cerebrovascular unit in the neurology department at Dokuz Eylül University Hospital.

First, neurologists were asked to evaluate the files according to the TOAST system within 60 days. Data files were delivered to neurologists by cargo in groups of ten in order not to confuse or rush them. Relevant articles regarding the TOAST classification system were also delivered within the first group. 

Next, evaluators registered with the web-based semiautomated CCS system at https://ccs.mgh.harvard.edu. They were certified after successfully completing 10 disease education modules including clinical and diagnostic tests offered by CCS version 2.0. Then neurologists were asked to evaluate the data files according to the CCS system. Files were randomized once again and delivered to the neurologists in groups of ten, together with relevant articles regarding the CCS classification system.

After all data were collected, each neurologist was assessed for compliance with the reference opinion according to both the TOAST and the CCS systems. In addition, neurologists were assessed in pairs for compliance and finally the concordance among all was calculated.

### 2.4. Statistical analysis 

Statistical analysis was performed using SPSS 15.0 for Windows. Diagnostic accuracy using different classification systems between the neurologists was determined by the kappa (ĸ) statistic. A ĸ-value of 0.80 and above was considered as excellent, 0.61–0.80 as strong, and 0.41–0.60 as moderate compliance. Each CCS system was categorized in 5, 8, and 16 categories to perform comparisons between groups. The Fleiss kappa method was used for multiple compliance analyses of more than two evaluators. 

## 3. Results

### 3.1. Clinical characteristics

Twenty out of 50 patients were women with a mean age of 70 (26–92) years. Risk factors for IS and demographic data are summarized in Table 2. Diffusion MRI could not be performed due to a pacemaker in one case and unstable vital findings in three cases. Vascular evaluation could not be performed due to unstable vital signs in one patient (2%). Stenosis was defined according to North American Symptomatic Carotid Endarterectomy Trial (NASCET) criteria as narrowing in the vessel lumen by at least 50%. Extracranial arteries were occluded in 4 cases (8%) and stenosis was found in 9 cases (18%). Intracranial arteries were occluded in 6 cases (12%) and stenosis was found in 4 cases (8%). The vascular evaluation of one venous infarct case revealed occlusion of the sinus rectus. Routine ECG was performed in all cases and cardiac arrhythmia was detected in 18. One patient was diagnosed with paroxysmal AF in rhythm Holter ECG. The risk of cardioembolism was high in 19 cases (38%) and low in 6 cases (12%).

**Table 2:** Demographic data of ischemic stroke patients.

Demographic data	n	%
Age (mean: 70, range: 26–92)		
Sex (female)	20	40
Risk factors		
Hypertension	33	66
Dyslipidemia	24	48
Cardiac arrhythmia	19	38
Diabetes mellitus	19	38
Cerebrovascular disease history	15	30
Smoking	23	46
Coronary artery/valvular heart disease	24	48
Cerebrovascular atherosclerosis	14	28
Hypercoagulability	5	10
Other (malignancy, family history of stroke, etc.)	6	12
Brain imaging		
Brain CT	50	100
Brain MRI	9	18
Diffusion MRI	46	92
Vascular imaging		
CT angiography	39	78
MRI angiography	5	10
DSA	5	10
Cervical Doppler	4	8
Infarct area		
Arterial infarct	49	98
Isolated ACA infarct	2	4
Isolated MCA infarct	21	42
Isolated PCA infarct	3	6
Isolated vertebrobasilar infarct	6	12
Multiple area infarcts	7	14
Border zone (watershed) infarcts	10	20
Venous infarct	1	2
Cardiac examinations		
ECG	50	100
Holter ECG	1	2
Transthoracic ECO	25	50
Transesophageal ECO	3	6

CT: Computerized tomography, MRI: magnetic resonance imaging, DSA: four system angiography, ASA: anterior cerebral artery, OSA: middle cerebral artery, PSA: posterior cerebral artery, ECG: electrocardiography, ECO: echocardiography.

### 3.2. TOAST and CCS comparison

The compliance of both stroke neurologists with the reference opinion was strong (ĸ: 0.77 and 0.67 for each neurologist) in the TOAST classification. On the contrary, their compliance was excellent for 5 subtypes in the CCS classification (ĸ: 0.83 and 0.86 for each; Table 3). The compliance of general neurologists with the reference opinion was also strong for the TOAST classification (ĸ: 0.76 and 0.78 for each). According to the CCS classification, compliance of the first neurologist was strong (ĸ: 0.70), while it was excellent for the second neurologist (ĸ: 0.89). 

**Table 3:** Compliance of evaluators according to TOAST and CCS subtypes.

		S1 vs. R	S2 vs. R	G1 vs. R	G2 vs. R	S1 vs. S2	G1 vs. G2	S vs. G
TOAST	K (95% CI)	0.77 (0.60–0.94)	0.67 (0.52–0.83)	0.76 (0.60–0.91)	0.78 (0.61–0.94)	0.61 (0.45–0.77)	0.64 (0.48–0.80)	0.59 (0.52–0.65)
CCS-5	K (95% CI)	0.83 (0.68–0.99)	0.86 (0.71–1.00)	0.70 (0.55–0.86)	0.89 (0.73–1.00)	0.78 (0.62–0.94)	0.75 (0.60–0.91)	0.75 (0.68–0.81)
CCS-8	K (95% CI)	0.84 (0.69–0.99)	0.84 (0.69–0.99)	0.71 (0.57–0.85)	0.86 (0.72–1.00)	0.73 (0.59–0.88)	0.74 (0.60–0.88)	0.73 (0.67–0.78)
CCS-16	K (95% CI)	0.68 (0.57–0.80)	0.65 (0.54–0.75)	0.55 (0.45–0.65)	0.78 (0.67–0.89)	0.52 (0.41–0.63)	0.53 (0.42–0.63)	0.53 (0.49–0.58)

K: Kappa value, CI: confidence interval, S: stroke neurologists, G: general neurologists, S1-S2: stroke neurologist 1-2, G1-G2: general neurologist 1-2, R: reference opinion.

Although the intrarater reliability was higher in the CCS than the TOAST system, the diagnostic compliance was still strong for both classification systems. When all four neurologists were considered, their compliance was moderate (ĸ: 0.59) in the TOAST system, whereas it was strong in the CCS system (ĸ: 0.75).

Subtype assignments for each neurologist were examined in the TOAST and CCS classifications (Table 4). The highest compliance was for ‘cardioembolism’ (91%) and ‘other identified causes’ (78.3%).

**Table 4:** According to TOAST and CCS classifications, the evaluators’ internal compliance, mean and median values.

	LAA	CE	SVO	OC	UE
	%	%	%	%	%
S1	75	90	0	66.7	83.3
S2	66.7	100	100	100	54.5
G1	70	89.5	33.3	100	58.3
G2	58.3	85.7	66.7	50	80
R	76.9	90	75	75	88.9
Average	69.9	91	55	78.3	73
Median	70	90	66.7	75	80

LAA, Large artery atherosclerosis; CE, cardioembolism; SVO, small vessel occlusion; OC, other causes; UE, undetermined etiology; S1-S2: stroke neurologist 1-2, G1-G2: general neurologist 1-2, R: reference opinion.

Table 5 summarizes the compliance rates including all 5 evaluations (2 stroke neurologists, 2 general neurologists, and the reference opinion) of 50 patients using 2 classification systems (500 evaluations in total). The rate of consensus (full compliance) was 44% in both TOAST and the CCS. The rate of full compliance with the CCS was 26% in cases where there was no consensus in TOAST. 

**Table 5:** Compliance analysis for subtyping according to TOAST and CCS.

Compliance analysis	n	%
Noncompliant in TOAST, full compliance in CSS Noncompliant in CSS, full compliance in TOAST Full compliance in TOAST and CSS No compliance in TOAST and CSS Increased TOAST compliance in CSS	13 2 22 11 2	26 4 44 22 4
Total	50	100

**Figure 1:**
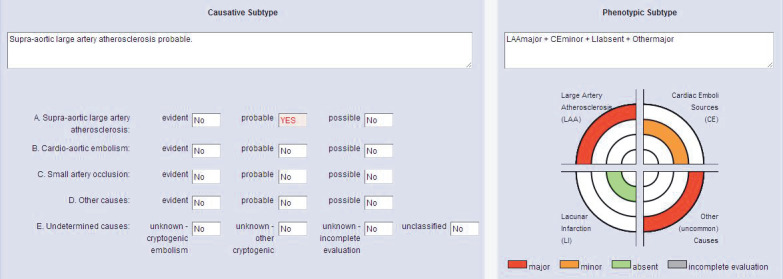
Etiologic and phenotypic subtype of stroke etiology according to CCS classification: as an example case in which language consensus between CCS and clinicians is provided is a 49-year-old patient, with internal border zone infarctions on the left, complete occlusion after left internal carotid artery bifurcation, mild SEK (spontaneous ECO contrast), heterozygous factor V Leiden mutation, and homozygous MTHFR A1298C mutation, which can be arguable. When assessed according to the TOAST classification (including the reference opinion), the three evaluators identified the etiologic cause of stroke as major arterial atherosclerosis. Two evaluators included the patient in the ‘unaccountable’ group due to the presence of two mechanisms (large artery atherosclerosis and other established causes). Later, when data were entered according to the CCS classification, two obvious causes (large artery atherosclerosis and other established causes) and a possible cause (cardioembolism) were detected. Language consensus was provided among researchers in the CCS, and five evaluators identified ‘large artery atherosclerosis’ as the stroke’s cause.

**Figure 2:**
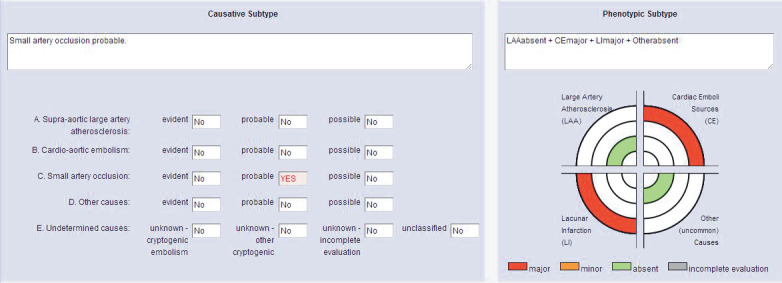
Etiologic and phenotypic subtype of stroke etiology according to CCS classification: a 92-year-old patient referred to the dysarthria-incompetent hand clinic with a lacunar infarct in the right corona radiata localization and atrial fibrillation was identified by an evaluator as having cardioembolism and one as small artery occlusion when assessed by TOAST (including reference opinion). The three evaluators made assignments that could not be determined by mentioning the possible presence of two possible mechanisms (cardioembolism and small artery occlusion) that could lead to a stroke. When the data were entered according to the CCS classification, two obvious causes (cardioembolism and small artery occlusion) were identified, but with the automated system in which the clinical features of the disease were included, the stroke cause was identified as ‘possible vascular disease’ by five evaluators and full compliance was achieved.

The total number of cases in the ‘undetermined etiology’ group was 73 (29.2%) in the TOAST classification and 54 (21.6%) in the CCS classification. The ‘undetermined etiology’ in TOAST decreased by 26% when the CCS system was used (Table 6) (this situation has been discussed in the context of patients in Figures 1 and 2). 

**Table 6:** Undetermined group analysis for subtyping according to TOAST and CCS.

	Undetermined group				
	TOAST		CCS		Change
	n	%	n	%	%
S1	17	34	12	24	29.4
S2	9	18	11	22	–22.2
G1	13	26	12	24	7.7
G2	19	38	10	20	47.3
R	15	30	9	18	40
Total	73	29.2	54	21.6	26.02

S1-S2: Stroke neurologist 1-2, G1-G2: general neurologist 1-2, R: reference opinion.

## 4. Discussion

In this study, the traditional TOAST system and the newer CCS system were used to investigate the concordance between stroke neurologists and general neurologists. In a study published by Ay et al. in 2007, the compliance rates of clinicians (neurologists specialized in stroke) were examined in all subgroups of the CCS (5). Perfect compliance for each group was obtained; however, the compliance rate decreased in parallel with the increasing number of subgroups. In the study conducted by Arsava et al. in 2010, 15 neurologists specialized in stroke, working in 13 centers in 8 countries, participated in the CCS reliability study, which reported ‘perfect’ compliance for CCS-5 subtypes, while compliance remained at the ‘strong’ level for CCS-8 and CCS-16 subtypes (6). In comparison with the 2007 study, the reduction in compliance in CCS subtype assignments could be explained by the reduction in the reliability ratio due to the increasing number of evaluators in kappa compliance analysis. High compliance in both studies might be due to the fact that all evaluations were performed between stroke neurologists. 

In this study, evaluators were examined in two categories. The compliance between two stroke neurologists was higher for CCS-5 and CCS-8 when compared to TOAST. Similarly, the compliance between two general neurologists was also higher in the CCS. In other words, using the CCS system after TOAST increased the compliance level from ‘strong’ to ‘close to perfect’ in both groups. It is not surprising that the CCS-16 subtype had lower compliance than CCS-5 and CCS-8 subtypes, as it is known that compliance decreases with the increasing number of options in accordance with the principles of kappa compliance analysis. 

When the compliance between all four evaluators was examined, the ĸ-value increased from 0.59 (TOAST) to 0.75 and 0.73 in the assignment of CCS-5 and CCS-8 subtypes, respectively. In other words, transition from moderate to strong compliance was achieved. In general, evaluations in pairs were reported in the literature in regards to the CCS system. In our study, although the quadruple evaluation showed lower compliance, ĸ-values still showed ‘strong compliance’ between pairs. There was 

no perfect compliance for any subtype of CCS, similar to the studies conducted in 2007 and 2010 (5,6).

In 2012, Lanfranconi et al. (14) published a study comparing the TOAST and CCS systems in which 690 patients were evaluated. According to this study, perfect compliance was found for both classification systems. However, the discriminating feature of this study was that a single evaluator tested the two systems and reported perfect compliance. This result reflects the fact that compliance will increase as the number of evaluators decreases. Similarly, in our study, when looking at the intrarater compliance in the two systems, compliance was found to be ‘strong’ and ‘strong, close to perfect’ for TOAST and CCS, respectively (ĸ: 0.62 and 0.78). 

The compliance of each stroke neurologist with the reference opinion in CCS-5 and CCS-8 subtypes was superior to that of the TOAST classification. The assessment of compliance of general neurologists with the reference opinion revealed excellent compliance of the first neurologist in CCS-5 and CCS-8 subtypes compared with TOAST. However, the second neurologist showed a lower compliance rate in subgroup assignments for CCS-5 and CCS-8. While the evaluator remained in strong compliance with the reference opinion in both systems, the ĸ-value was 0.76 in TOAST and 0.70 in the CCS-5 system. Analyses revealed that the major cause of the mistakes was the lack of entry of the identified etiological information into the system.

Both in TOAST and the CCS, interrater compliance was reviewed according to the main subgroups. The highest compliance was detected in subtypes of ‘cardioembolism’ and ‘other identified causes’. Given the fact that treatment of cardioembolic stroke and other established causes differs from other categories, one of the advantages of the CCS system would be the better recognition of these subtypes. Unlike our study, the ‘undetermined etiology’ group and ‘cardioembolism’ were reported as the most frequent etiologies in the studies of Lanfranconi et al. and a prospective cohort study in North Dublin (10,14). Moreover, in the NINDS SiGN study, which was a pooled analysis of 20 studies that enrolled 13,596 patients, the highest compliance between the evaluators was in ‘great artery atherosclerosis’ while the lowest compliance was found in ‘small artery occlusion’ (7). 

One of the main objectives of the CCS classification is to reduce the number of patients that cannot be identified in TOAST due to multiple etiologies (2,4–6). In 2005, Ay et al. compared TOAST with the SSS-TOAST system, the ancestor of CCS, and reported a decrease in the ‘undetermined etiology’ group from 38%–40% to 4% (4). Likewise, Arsava et al. reported that 22% of ischemic stroke cases had multiple etiologies; however, this rate was between 0% and 8% with the CCS (6). Classification with the CCS decreased the number of the ‘undetermined etiology’ group in the North Dublin study by 33.3% (10) and in Gökcal et al.’s study by 26% (15) when compared with TOAST. Arsava et al.’s study (1816 patients included) in 2017 also reported that the size of the undetermined category was 33% by the CCS and 53% by TOAST (16). On the contrary, Lanfranconi et al. reported that 35 out of the 204 cases that were unaccountable with TOAST had been assigned to a subtype in the CCS, whereas 32 out of 200 cases without a definite etiology with the CCS had been assigned to a subtype with TOAST (14). In conclusion, they stated that there was no significant difference in the ‘undetermined etiology’ group between TOAST and the CCS. In our study, a 26% reduction was detected in CCS classification compared with TOAST in the proportion of the ‘undetermined etiology’ group.

As a result, the CCS system improved compliance both in stroke neurologists and general neurologists when compared to TOAST in the classification of ischemic stroke. In addition, there was a decline in the proportion of unexplained cases in the CCS classification. The highest compliance was detected in subtypes of ‘cardioembolism’ and stroke due to ‘other identified causes’.

This study has some major limitations. First, we have a small sample size, which limited our statistical power to evaluate the agreement between the classification systems studied. However, this study has made a major improvement in the etiological classification of ischemic stroke compared to the stroke neurologist and general neurologist. The other limitation of our study is that not all patients were evaluated by echocardiography and 24-h Holter ECG monitoring, which might have limited the identification of cardioembolism.

In conclusion, correct identification of ischemic stroke subtype is the most important issue in approaching stroke patients, both for medical management and in ensuring language consensus among researchers in multicenter clinical trials. This study made a major innovation in the comparison of stroke neurologists and general neurologists, as well as contributing the data of a stroke unit from Turkey to the literature. The results of this study suggest that the automatic, evidence-based, and easily reproducible CCS system could be superior to the TOAST system for accurate subtyping. The CCS could be recommended in both routine clinical management of stroke patients and patient selection in multicenter clinical trials instead of TOAST.
